# The Impact of Prostate Imaging Reporting and Data System Version 2.1 and Prostate-Specific Antigen Density in the Prediction of Clinically Significant Prostate Cancer

**DOI:** 10.5152/tud.2023.220199

**Published:** 2023-03-01

**Authors:** Sehnaz Tezcan, Funda Ulu Ozturk, Ulku Bekar, Erdem Ozturk

**Affiliations:** 1 Department of Radiology, Koru Hospital, Ankara, Turkey; 2 Department of Radiology, Başkent University Hospital, Ankara, Turkey; 3 Department of Urology, Dr. Abdurrahman Yurtaslan Ankara Oncology Training and Research Hospital, Ankara, Turkey

**Keywords:** Prostatic neoplasms, magnetic resonance imaging, prostate-specific antigen

## Abstract

**Objective:**

The aim of this study was to evaluate the diagnostic performance of multiparametric magnetic resonance imaging for clinically significant prostate cancer and to determine whether applying Prostate Imaging Reporting and Data Systems version 2.1 score could improve the diagnostic pathway besides the biochemical characteristics.

**Materials and methods:**

In this study, 199 patients with clinically suspected prostate cancer who underwent multiparametric magnetic resonance imaging were included. Logistic regression analyses and receiver operating characteristic curve were performed to determine independent predictors and to compare diagnostic performance of indicators for clinically significant prostate cancer. Two models were established. In model 1, the diagnostic performance of prostate-specific antigen- and prostate-specific antigen density-derived parameters were evaluated. In model 2, the prediction potential of model 1 plus Prostate Imaging Reporting and Data Systems version 2.1 score was analyzed.

**Results:**

Sixty-four patients were positive for clinically significant prostate cancer by histopathological analysis (32.1%). In model 1, a prostate-specific antigen density >0.15 was labeled as the strongest predictor of malignancy. In model 2, a prostate-specific antigen density >0.15, a Prostate Imaging Reporting and Data Systems score ≥3, and a Prostate Imaging Reporting and Data Systems score ≥4 demonstrated the strongest association with malignancy. Among these parameters, a Prostate Imaging Reporting and Data Systems score ≥4 (*P* = .003) was found to be the most robust predictor for malignancy, followed by a Prostate Imaging Reporting and Data Systems score ≥3 (*P* = .012). The multivariate analysis revealed higher accuracy in model 2 (76.9%) than in model 1 (67.8%). The area under curve values with respect to prostate-specific antigen, prostate-specific antigen density, model 1, and model 2 were 0.632, 0.741, 0.656, and 0.798, respectively.

**Conclusion:**

These results indicated that Prostate Imaging Reporting and Data Systems version 2.1 score and prostate-specific antigen density are independent predictors for the presence of clinically significant prostate cancer. Both prostate-specific antigen density and Prostate Imaging Reporting and Data Systems version 2.1 score should be risen to prominence in the decision of biopsy instead of PSA.

**Main Points**

- Serum prostate-specific antigen (PSA) is a main method in the screening of clinically significant prostate cancer. Despite high sensitivity of PSA, the low specificity results in unnecessary biopsy procedures. In recent years, prostate-specific antigen density (PSAD) has become a widely used biomarker to enhance the diagnostic performance of PSA. Although the different cutoff values of PSAD have been studied in many previous studies, the threshold of 0.15 ng/mL/cc is the most accepted cutoff, with more accurate results in the literature.
- Multiparametric prostate magnetic resonance imaging (mpMRI) has gained an essential role in the detection of clinically significant prostate cancer. The use of Prostate Imaging Reporting and Data Systems (PI-RADS) has increased the confidence in MRI. Prostate Imaging Reporting and Data Systems version 2.1 (PI-RADSv2.1) has been developed in 2019 which is the current form of the PI-RADS.
- Prostate-specific antigen density and mpMRI showed more accurate results than serum PSA levels in the diagnosis of clinically significant prostate cancer. Applying the PI-RADSv2.1 score to the biochemical characteristics increased the probability of the detection of prostate cancer. The PI-RADSv2.1 score and PSAD are both independent and strong predictors for the presence of clinically significant prostate cancer.
- In the decision of biopsy, the urologists should pay attention to mpMRI findings and PSAD instead of PSA in clinical practice.

## Introduction

Prostate cancer (PC) is the most frequently diagnosed disease among men worldwide. Prostate-specific antigen (PSA) is the main screening method to detect PC.^1^ Currently, the PSA cutoff value of 4 ng/mL has been used to perform prostate biopsy.^2^ The high serum PSA levels in patients with benign conditions may cause unnecessary biopsy procedures. The prostate-specific antigen density (PSAD) has become a commonly used biomarker to enhance the accuracy of PSA.^3^ The PSAD threshold of 0.15 ng/mL/cc, particularly in cases of high PSA levels and negative multiparametric magnetic resonance imaging (mpMRI) results, has been suggested by Prostate Imaging Reporting and Data Systems (PI-RADS) version (v) 2.1 for the biopsy decision.^4,5^ Recent studies underlined that PSAD was a more accurate predictor of PC, having similar or better sensitivity but greater specificity compared to PSA levels.^6-8^

In recent years, mpMRI has become a widely used modality for the detection of clinically significant (CS) PC prior to biopsy.^5,9,10^ The PI-RADSv1 was published in 2012. The PI-RADSv2 was published in 2015 to improve inter-observer agreement (IOA) and accuracy of prior PI-RADS by the American College of Radiology and European Society of Urogenital Radiology.^5^ The majority of previous studies reporting the validity of PI-RADSv2 showed fair to substantial agreement among radiologists in the diagnosis of CSPC.^5,11-13^ To enhance PI-RADSv2, PI-RADSv2.1 has been developed in 2019 which is the current form of the PI-RADS.^5^

To the best of our knowledge, few published studies have demonstrated the importance of PI-RADSv2.1 score and laboratory indicators in the determination of CSPC.^14-16^ The purpose of this study was to evaluate the diagnostic performance of mpMRI and PSA-based parameters in the diagnosis of CSPC.

## Materials and Methods

### Study Population

The study was approved by the Ethics Committee of Dr. Abdurrahman Yurtaslan Ankara Oncology Training and Research Hospital (Approval No: 2022-02/1646). All procedures performed in this study involving human participants were in accordance with the 1964 Declaration of Helsinki. The written informed consent was obtained from all participants who participated in this study. Five hundred forty-one patients with clinically suspected PC based on PSA or clinical examination who underwent mpMRI (1.5 Tesla) between January 2017 and January 2022 were enrolled in this study. Patients were excluded due to the absence of histopathological results and valid PSA levels. One hundred ninety-nine patients who underwent systematic 12-core trans-rectal ultrasound (TRUS)-guided biopsy were finally included in this study. Patients were categorized into groups regarding the PSA, PSAD levels, age, and PI-RADSv2.1 scores (PI-RADS ≥ 3 and PI-RADS ≥ 4). All MRI examinations were evaluated by 2 experienced radiologists who were blinded to patients’ data. The International Society of Urological Pathology classification was used to categorize the lesions. The International Society of Urological Pathology grade ≥2 was defined as CSPC. All specimens were evaluated by an experienced pathologist.

### Magnetic Resonance Imaging Protocol

All MRI examinations were performed using 1.5 T MRI with an 8-channel body/torso array coil. All patients were examined in the supine position. A routine protocol was performed, including T2-weighted imaging (T2WI), diffusion-weighted imaging (DWI) with apparent diffusion coefficient (ADC) map, T2 fat-sat, T1WI, and dynamic contrast-enhanced (DCE) images. The DCE images were obtained after administration of 0.1 mmol/kg of gadoteric acid. Diffusion-weighted imaging was performed using *b* values of 50, 1000, and 1400 s/mm^2^. The MR image acquisition protocol was as follows: axial T2WI sequence [repetition time (TR), 5594 ms; echo time (TE), 90 ms; slice thickness, 3 mm; field of view (FOV), 20 × 20 mm^2^] and sagittal T2WI sequence (TR, 4300 ms; TE, 102 ms; slice thickness, 3 mm; FOV, 24 × 24 mm^2^). The axial DWI sequence (TR, 5400 ms; TE, 80 ms; slice thickness, 3 mm; FOV, 20 × 20 mm^2^) had multiple *b* values (*b* = 0, 1000, and 1400 s/mm^2^). Apparent diffusion coefficient maps were obtained from *b* = 1000 and *b* = 1400 s/mm^2^.

### Statistical Analysis

The Kolmogorov–Smirnov test was used to analyze the normal distribution of data. The variables were compared using the Mann–Whitney *U*-test or Student’s *t*-test. The *P*-value less than .05 was considered to show a significant difference. Univariate and multivariate binary logistic regression analyses were performed to determine the significant indicators. We established 2 models. In model 1, we analyzed the parameters of PSA > 4 ng/mL, PSAD > 0.15 ng/mL/cc, PSAD > 0.30 ng/mL/cc, age > 50, age > 60, and age > 70 years. In model 2, we added PI-RADS ≥ 3 and PI-RADS ≥ 4 to model 1. A receiver operating characteristic (ROC) analysis was used to estimate the area under curve (AUC) of all predictors. The IOA between each reader for lesions was evaluated by using Cohen’s weighted kappa statistics, considering categories according to Landis and Koch recommendations [Kappa (*K*) value; <0 poor; 0.00-0.20 slight; 0.21-0.40 fair; 0.41-0.60 moderate; 0.61-0.80 substantial; 0.81-1.00 almost perfect) with 95% CIs. Analyses were performed using Statistical Package of Social Sciences Version 22.0. (IBM SPSS Corp.; Armonk, NY, USA).

## Results

The characteristics of patients are demonstrated in Table 1. The total study population was 199 patients, including 64 (32.1%) patients with CSPC and 135 (67.8%) patients with benign pathology. The age, total PSA, and PSAD of the malignant group were significantly higher than that of the benign group (*P* < .001). The prostate volume of the malignant group was significantly lower than the benign group (*P* < .001).

The *K* value of the IOA was higher in PI-RADS ≥ 4 (*K* value, 0.672; *P* < .001) than PI-RADS ≥ 3 (*K* value, 0.625; *P* < .001). While the sensitivity of PI-RADS ≥ 3 was higher than PI-RADS ≥ 4 (92.1% vs. 71.8%; *P* <.001), the specificity was higher in PI-RADS ≥ 4 (48.1% vs. 74.8%; *P* < .001). The sensitivity and specificity of PSA > 4 were 92.1% and 11.1%, respectively. The sensitivity and specificity of PSAD > 0.15 ng/mL/cc were 59.3% and 71.8%, respectively. The sensitivity and specificity of PSAD > 0.30 ng/mL/cc were 29.6% and 91.8%, respectively.

In univariate analysis, while PSAD > 0.15 ng/mL/cc, PSAD > 0.30 ng/mL/cc, age > 70, PI-RADS ≥ 3, and PI-RADS ≥ 4 were significantly associated with the presence of CSPC (*P* < 0.05), there was no association for PSA > 4 ng/mL, age > 50, and age > 60 (Table 2). In model 1, PSAD > 0.15 ng/mL/cc was the strongest predictor of malignancy. In model 2, PSAD > 0.15 ng/mL/cc, PI-RADS ≥ 3, and PI-RADS ≥ 4 were all the discriminators of CSPC, where PI-RADS ≥ 4 was the strongest predictor, followed by PI-RADS ≥ 3 (Table 2). The multivariate analysis demonstrated higher accuracy in model 2 compared to model 1. The overall percentage rates of model 1 and model 2 were 67.8% and 76.9%, respectively.

**Figure 1.**
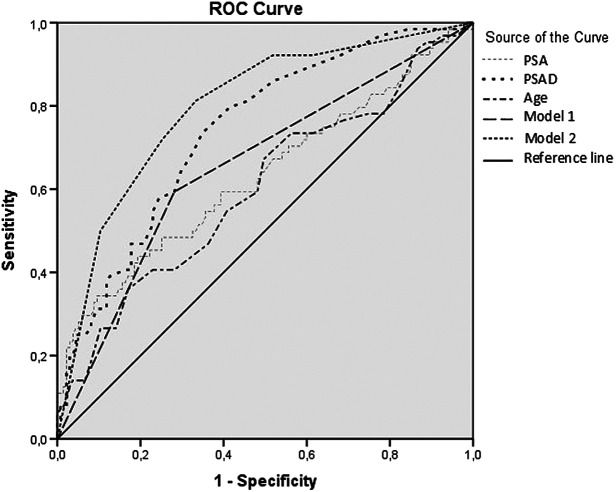
ROC curves for the PSA, PSAD, age and 2 established models in detecting clinically significant prostate cancer. PSA, prostate-specific antigen; PSAD, prostate-specific antigen density; ROC, receiver operating characteristic.

The ROC analysis data, including AUC values, of the independent indicators and prediction models for CSPC are shown in Table 3 and Figure 1. Among clinical and laboratory parameters, PSAD had the highest AUC for predicting CSPC. Model 2 showed the highest AUC (AUC = 0.798, *P* < .000), which was significantly different from model 1 (AUC: 0.656, *P* = .01) and other single indicators.

## Discussion

Currently, screening for PC remains a controversial issue in urology. The use of PSA levels is the main screening method of PC. However, several studies showed no correlation between elevated PSA levels and PC.^17^ Benign conditions may result in PSA increase, as well. Despite high sensitivity rates of PSA levels in the diagnosis of PC, the low specificity rates yield to unnecessary biopsy procedures.^6-8^ The PSA cutoff value of 4 ng/ml is considered as a common threshold for the biopsy decision.^2^ In the current study, the cutoff value of 4 ng/mL demonstrated high sensitivity and low specificity in the diagnosis of CSPC. When PSA > 4 ng/mL was used as the cutoff, the unnecessary biopsy rates increased, but the risk of missing malignancy reduced. Akdogan et al^18^ found the sensitivity and specificity of PSA cutoff value of 4 ng/mL to be 89.6%, and 15.7%, respectively, which is consistent with our study. Although recent PC screening is still based on serum PSA levels, low specificity and false positivity increased the popularity of PSA-derived parameters, such as PSAD.

Prostate-specific antigen density is based on PSA levels and prostate volume. In the literature, higher accuracy results were found for PSAD in the diagnosis of CSPC.^14-17,19^ Verma et al^7^ found that PSAD (AUC = 0.72) was more accurate than PSA (AUC = 0.61). They also reported a sensitivity and specificity of 68% and 66% at a cutoff value of 0.15 for PSAD, which is similar to our study.^7^ We obtained higher sensitivity and lower specificity when PSAD > 0.15 is assigned as the cutoff compared to PSAD > 0.30. A recent study showed that lowering the PSAD cutoff to 0.08 provided an increase in negative predictive value to 96%.^9^ Another study revealed that in patients with negative mpMRI, establishing PSAD > 0.10 as the cutoff resulted in the decrease of unnecessary biopsies while still catching malignancy at the most.^20^ In the current study, apart from the mpMRI findings, PSAD > 0.15 was found the strongest independent predictor of CSPC, followed by age. In the decision of biopsy, the PSAD should come to the forefront instead of PSA levels.

In the literature, AUC for PSAD ranges from 0.65 to 0.75.^8,14,16,19^ Our results are similar, where PSAD demonstrates higher AUC than the PSA and age.^14-16,19,21^ The strength of PSAD in the diagnosis of CSPC underlines the importance of the prostate volume measurement. Prostate Imaging Reporting and Data Systems version 2.1 includes a change in the method for calculating the prostate volume. While the mid-axial plane is recommended in PI-RADSv2, it is switched to the mid-sagittal plane in PI-RADSv2.1 in order to measure the anteroposterior diameter of the prostate.^5^ Gündoğdu et al^22^ showed better reproducibility results for PI-RADSv2.1 compared to PI-RADSv2, in the measurement of prostate volume. Accurate assessment of prostatic volume is necessary in the planning for treatment and also for the calculation of PSAD. Although the volume measurement can be performed by TRUS, mpMRI has the advantages of high resolution and not being operator dependent.

In recent years, mpMRI has gained an important role in the diagnostic algorithm of PC due to the widespread use of PI-RADS. The results from a recent meta-analysis revealed that the sensitivity and specificity of PI-RADSv2.1 for diagnosing CSPC were 87% and 74%, respectively.^23^ Besides, the pooled sensitivity and specificity of PI-RADSv2.1 for a cutoff of PI-RADS≥4 were 81% and 82%, respectively, and the sensitivity and specificity of PI-RADSv2.1 for a cutoff of PI-RADS ≥ 3 were 94% and 56%, respectively.^23^ Our study showed that the sensitivity and specificity of PI-RADSv2.1 were 71.8% and 74.8%, respectively, for PI-RADS ≥ 4 and 92.1% and 48.1%, respectively, for PI-RADS ≥ 3. The relatively lower results in our study may be related to the use of 3T MRI system in the meta-analysis. We found a substantial agreement of PI-RADSv2.1 category assessment among readers. The majority of previous studies revealed low-to-moderate agreement of PI-RADSv2 among readers, while a few reports showed substantial agreement.^11,23,25^ We found higher IOA for PI-RADSv2.1 compared to similar past studies evaluating PI-RADSv2.^24,25^

The studies evaluating the predictors of CSPC agreed that PI-RADSv2.1 score is a stronger indicator of malignancy than PSA and PSAD.^2,14-16^ Our results are consistent with past studies. A previous study by Han et al^16^ evaluating the performance of mpMRI, PSAD, and a combined model including both demonstrated that the combined model shows better performance (AUC = 0.682, 0.867, and 0.896 for PSAD, mpMRI, and combined model, respectively) for cancer diagnosis in patients with PSA levels of 4-10 ng/mL. In our study, we found that adding PSAD and mpMRI has increased the predictive potential of the PSA for CSPC diagnosis, which is consistent with the study of Han et al.^16^

Prostate-specific antigen is a main method in the screening of CSPC worldwide. When using PSA alone in the decision of biopsy, the probability of accurate diagnosis may decrease as mentioned in the literature. Adding mpMRI and PSAD may provide better diagnostic performance while reducing unnecessary biopsy procedures. Also, the predictive potential of PI-RADS is not directly affected by prostate volume as it is with PSAD. Moreover, predicting the index lesion with mpMRI prior to TRUS biopsy procedure may be helpful to improve the diagnostic performance via focusing on the most suspicious core for multiple tissue sampling. The mpMRI and PSAD should be adapted mostly to the diagnostic pathway due to the better predictive potential.

Our study had some limitations. The study design was retrospective. All examinations were performed at a single center with the same protocol which could induce similar approaches. Additionally, in our study, the pathological results were obtained from TRUS-guided systematic prostate biopsy, whereas the use of MR-guided prostate biopsy may increase the detection rate of CSPC. Further studies with the pathological analysis based on the MRI-targeted biopsy are needed in the evaluation of the diagnostic performance of MRI and the biochemical parameters. Also, our study was limited by the absence of whole-mount histologic correlation. Hence, the pathological analysis may be less reliable considering the lack of evaluating all segments of the prostate tissue. On the other hand, one strength of our study is the use of newly described version of PI-RADS classification which was discussed in a few previous studies.

In conclusion, PSAD and mpMRI revealed more reliable results than serum total PSA levels for the prediction of malignancy. Our results showed that the PI-RADSv2.1 score combined with other clinical parameters showed a robust predictive potential for CSPC diagnosis. The PI-RADSv2.1 score and PSAD are both significant and independent predictors for the presence of CSPC. The urologists should give more preference to PSAD and mpMRI findings in the decision of biopsy instead of PSA levels.

## Figures and Tables

**Table 1.** Patient Characteristics

	Cancer Group, Mean ± SD (IQR: 25th, 50th, 75th)	Non-cancer Group, Mean ± SD (IQR: 25th, 50th, 75th)	*P*
Patients, n	64	135	
Age (years)	66.2 ± 7.8 (61, 66, 72)	63.3 ± 7 (59, 63, 68)	**.01**
Total PSA (ng/mL)	20.2 ± 38.5 (5.3, 7.6, 17.1)	7.6 ± 5.1 (4.7, 6.1, 8.6)	**.003**
PSAD (ng/mL/cc)	0.39 ± 0.63 (0.12, 0.19, 0.35)	0.15 ± 0.2 (0.07, 0.10, 0.16)	**<.000**
Prostatic volume (cc)	51.6 ± 27.9 (37.9, 44.2, 58.9)	77.3 ± 57 (41.7, 63.3, 87.8)	**<.000**
ISUP (n)			
2	23		
3	21		
4	12		
5	8		

IQR, interquartile range; ISUP, International Society of Urological Pathology; PSA, prostate-specific antigen; PSAD, prostate-specific antigen density.

*P*-value < .05 was set as statistically significant. *Statistically significant; *P*-values are expressed in Bold.

**Table 2.** Univariate and Multivariate Analysis of Variables

	Univariate Analysis		Multivariate Analysis			
			Model 1		Model 2	
	ExpB (OR) (95% CI)	*P*	ExpB (OR) (95% CI)	*P*	ExpB (OR) (95% CI)	*P*
PSA > 4 (ng/mL)	1.475 (0.512-4.253)	.472				
PSAD > 0.15 (ng/mL/cc)	3.731 (1.999-6.964)	**<.000**	3.731 (1.999-6.964)	**<.000**	2.181 (1.086-4.381)	**.028**
PSAD > 0.30 (ng/mL/cc)	4.760 (2.102-10.776)	**<.000**				
Age > 50	1.192 (0.225-6.318)	.836				
Age > 60	1.425 (0.718-2.826)	.311				
Age > 70	2.208 (1.057-4.614)	**.035**				
PI-RADS ≥ 3	10.957 (4.140-29.001)	**<.000**			4.191 (1.366-12.855)	**.012**
PI-RADS ≥ 4	7.592 (3.887-14.825)	**<.000**			3.268 (1.482-7.208)	**.003**

PI-RADS, Prostate Imaging Reporting and Data Systems; PSA, prostate-specific antigen; PSAD, prostate-specific antigen density.

*P*-value < .05 was set as statistically significant. *Statistically significant; *P*-values are expressed in Bold.

**Table 3.** Receiver Operating Characteristics for the Prediction of Clinically Significant Cancer

Predictor	Area under curve	Standard error	95% CI	*P*
PSA (ng/mL)	0.632	0.045	0.544-0.720	.003
PSAD (ng/mL/cc)	0.741	0.037	0.669-0.812	<.000
Age	0.602	0.044	0.515-0.689	.02
Model 1	0.656	0.042	0.573-0.739	<.000
Model 2	0.798	0.034	0.732-0.865	<.000

PSA, prostate-specific antigen; PSAD, prostate-specific antigen density.
